# Determining suspended solids and total phosphorus from turbidity: comparison of high-frequency sampling with conventional monitoring methods

**DOI:** 10.1007/s10661-019-7775-7

**Published:** 2019-09-04

**Authors:** Ana Villa, Jens Fölster, Katarina Kyllmar

**Affiliations:** 1 Department of Soil and Environment, Swedish University of Agricultural Sciences, P.O. Box 7014, 75007 Uppsala, Sweden; 2 Department of Aquatic Sciences and Assessment, Swedish University of Agricultural Sciences, P.O. Box 7050, 75007 Uppsala, Sweden

**Keywords:** Turbidity, Suspended solids, High-frequency sensor, Phosphorus transport

## Abstract

Suspended solids (SS) are important carriers of pollutants such as phosphorus (P) in streams, but the sampling frequency in monitoring programs is usually insufficiently frequent to capture episodic SS and total P (TP) peaks. The suitability of turbidity and conductivity as a surrogate for SS and TP was studied using 108 monitoring stations located in catchments of different sizes, land uses, and pollution levels. The use of high-frequency turbidity measurements to estimate SS and TP loads was compared with the use of two sampling methods (grab, flow-proportional sampling) in a case study. When all samples were considered, turbidity was a good predictor of SS (*r*^2^ = 0.76) and TP (*r*^2^ = 0.75). For single sites, there was a large range in how well turbidity could predict the two variables. The site-specific turbidity-SS relationship was significant at 87% of sites (mean *r*^2^ = 0.72). The site turbidity and conductivity-TP relationship was significant at 78% of sites (mean *r*^2^ = 0.62). A stronger turbidity-SS relationship was found in catchments with a higher percentage of agricultural land. The turbidity and conductivity-TP relationship was stronger when the TP concentration was high. In the case study, TP loads were smallest when estimated with grab sampling, which missed several discharge peaks. Loads estimated with high-frequency turbidity measurements were 19–51% smaller than with flow-proportional sampling, probably due to differences in sampling points. High-frequency turbidity measurements can be a viable alternative to conventional sampling methods in studies on concentration dynamics and load estimates.

## Introduction

Eutrophication caused by excessive nutrient concentrations and loads in surface waters is affecting the Baltic Sea (Karlson et al. 2002) and many other water bodies around the world (Smith 2003). In response to this growing problem, measures aiming at restoring affected water bodies and controlling nutrient transport have been introduced (Sharpley et al. 2000; Hart 2003; Schutz et al. 2017). To evaluate the effectiveness of mitigation measures, proper monitoring of surface waters is needed to classify water status accurately, detect trends over time, and estimate pollutant loads discharging into water bodies. These objectives are specified in European Union legislation, e.g., the Water Framework Directive (WFD) (2000/60/EC), and political bodies, e.g., the Commission for the Protection of the Marine Environment in the Baltic Sea Area (HELCOM 2013).

Given the variation in water quality in space and time, the representativeness of samples is a critical issue when selecting the most appropriate monitoring strategy and frequency. To date, the most common monitoring frequency has been monthly or fortnightly grab sampling (Ferreira et al. 2007). This can be sufficient for some indicators, contaminants, or purposes, but not others, since, e.g., Skeffington et al. (2015) showed that, using monthly sampling, a particular water body could be described as having good, moderate, or poor ecological status due to random sampling effects. “Good Ecological Status” is the WFD default objective for all water bodies and is defined as only slight variation from undisturbed conditions. Monthly or fortnightly grab sampling often misses short high-concentration peaks in suspended solids (SS) and total phosphorus (TP) (Brauer et al. 2009), which typically originate from small areas of a catchment (Sharpley et al. 2009) and occur during a few high-flow episodes per year (Bowes et al. 2003; Horowitz 2008). Decreasing sampling frequency increases the variability and uncertainty in annual load estimates (Bowes et al. 2009; Bieroza et al. 2018). Cassidy and Jordan (2011) found that only near-continuous monitoring (e.g., hourly and sub-hourly sampling) is able to capture the large temporal changes in TP concentrations in small agricultural streams. More frequent measurements of SS or TP concentrations than those used in conventional monitoring are needed to reduce the uncertainty in long-term load estimates, especially in flashy streams (Jones et al. 2012) and in streams with large losses of SS and TP. Composite sampling, such as flow-proportional sampling, is another strategy used to improve load estimates and have been explored and integrated in agricultural monitoring catchments in the Nordic and Baltic countries (Kyllmar et al. 2014a). Cassidy et al. (2018) showed that load estimates based on flow-proportional composite sampling were accurate when compared against estimates from daily and sub-daily discrete sampling. However, while flow-proportional sampling improves the accuracy of load estimates, it does not describe temporal or spatial variations in water quality. In practice, an increase in frequency to weekly or daily sampling is not always possible, due to high costs and resource constraints. In the selection of a sampling strategy, costs and accuracy need to be balanced when seeking to obtain a proper representation of concentrations and loads (Brauer et al. 2009).

High-frequency measurement with sensors is an increasingly common sampling option (Rode et al. 2016). It is seen as more cost-effective, allowing a greater number of measurements to be made compared with conventional grab sampling regimes (Davies-Colley and Smith 2001). Collection of high-frequency data at multiple sites has been proven to quantify effectively the spatial and temporal variation in water quality constituent fluxes (Horsburgh et al. 2010). However, constituents such as SS and P cannot be measured with current in situ sensors and surrogate measures to estimate them are needed. Turbidity has been widely used as a surrogate to predict concentrations of suspended particles (Bilotta and Brazier 2008). It is a relative measure of light diffraction in water caused by suspended particles. The use of turbidity as a surrogate for TP is based on the premise that most of the TP transported in streams is in particulate form (Settle et al. 2007; Stubblefield et al. 2007; Ruegner et al. 2013). While the relationship between turbidity and SS may be confounded by factors such as variations in particle size distribution, particle composition, or water color (Ruzycki et al. 2014; Bright et al. 2018), a certain level of error can be tolerated, as continuous estimation of SS concentrations overcomes the greatest error, which derives from infrequent sampling (Gippel 1995). High-frequency sensor surrogate measurements can be expected to reduce the uncertainty in load estimation by accounting for variations in concentrations and can produce similar results for SS and TP as continuous sampling (e.g., hourly, daily), which can be considered to represent the *true* loads. Conductivity is another surrogate parameter that has been widely used, as it is easy and inexpensive to measure. Continuous conductivity measurements have served for hydrograph separation (Heppell and Chapman 2006; Pellerin et al. 2008; Lange and Haensler 2012) and could potential complement turbidity as a surrogate of TP concentration measurements when there are sources of P of different hydrological origins.

Most previous studies involving the use of surrogates to estimate SS and TP concentrations have focused on a few heavily monitored sites, while only a few studies have used multiple sites. Recently, Schilling et al. (2017) evaluated 43 different river monitoring sites in Iowa and found that TP concentrations were highly correlated to turbidity, with a mean correlation coefficient (*r*) of 0.78. Davies-Colley et al. (2014) used 77 diverse New Zealand rivers to explore the relationships between SS, turbidity, and visual clarity and found that SS and turbidity were highly correlated (*r*^2^ = 0.87) when all samples from the 77 sites for a 12-month period were pooled. The standard error (SE) was also high (0.313) in that study, meaning that the relationship was not precisely predictive, possibly due to the large variation between individual sites. For individual sites, they found lower SE values, suggesting that turbidity could be used as a surrogate for SS when site-specific relationships are established (Davies-Colley et al. 2014). Grayson et al. (1996) evaluated the use of general relationships with pooled data from sites throughout a 5000-km^2^ catchment, under a wide range of flow conditions and were able to explain 70–90% of the variation in the relationships despite a wide range of flow, climate, and catchment conditions. Discharge-specific regressions are reported to show higher *r*^2^ values than those based on pooled data (Pfannkuche and Schmidt 2003; Lacour et al. 2009). However, the general relationships in those studies still showed *r*^2^ values above 0.8. Stutter et al. (2017) found that the correlations between turbidity and TP or SS are always site-specific and thus non-transferable between catchments.

In the present study, we explored the use of surrogates (turbidity and conductivity) for SS and TP concentrations in an extensive dataset of 108 sites across Sweden. The sites ranged from first-order rivers (which cover the entire catchment to the estuary) to small headwater streams and catchments with various land uses (from pristine forest catchments to heavily polluted agricultural catchments). The use of high-frequency measurements of surrogate parameters is of particular interest in Sweden, where over 90% of the total length of perennial streams (530,000 km) is associated with a catchment area of less than 15 km^2^ (Bishop et al. 2008). Many headwater catchments are currently not being monitored, as doing so with the current sampling method (i.e., grab sampling) would not be feasible due to the large number of water bodies involved. The use of sensors to measure proxy parameters could help extend the number of monitored sites and improve estimation of SS and TP concentrations and loads, especially since lower-cost, robust sensors for long-term field deployment are becoming more readily available. However, before making use of proxy turbidity measurements, the relationship between turbidity and SS and TP needs to be determined in a number of streams.

The aims of this study were thus (1) to evaluate the use of turbidity as a surrogate for SS and TP concentrations at 108 river monitoring sites in Sweden, using general and site-specific relationships, and (2) to compare TP load estimates obtained using high-frequency sensor measurements, grab sampling, and flow-proportional sampling.

## Materials and methods

### Study areas and sampling strategy

**Fig. 1:**
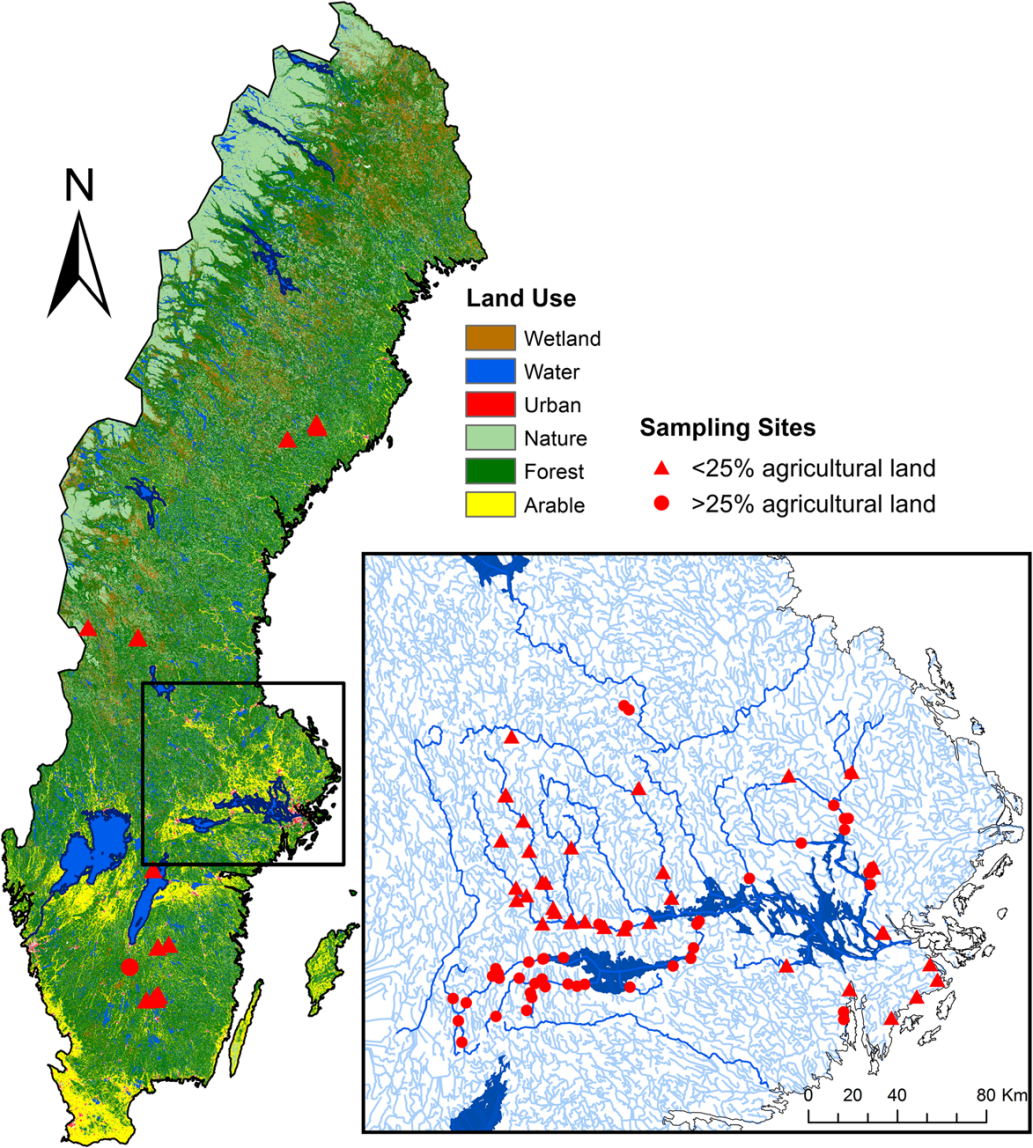
Map showing the location of the sampling sites and land use over Sweden. Triangles represent sites located in catchments with < 25% agricultural land, while circles are sites in catchments with > 25% agricultural land. The region with a higher density of samples is enlarged and includes a representation of streams

The analysis was performed on a set of 108 stream monitoring stations included in national and regional monitoring programs, research projects, and the “coordinated recipient monitoring” program (*Recipientkontroll* (*SKK och RK*) in Swedish). The latter is the statutory monitoring program conducted by dischargers of pollutants (e.g., private companies, municipalities) downstream of the discharge site. The study sites were distributed across different regions in Sweden, from latitude 57° N to 64° N, covering a wide range of catchment sizes, land uses, and pollution levels (Fig. 1). Catchment size ranged from 0.24 to 4182.4 km^2^ (median 110.3 km^2^), which included sites ranging from small tributary streams to first-order rivers. The most extensive land use in the catchments was forestry (median 72%), followed by agriculture (median 9%). The latter was mainly concentrated in southern Sweden (Fig. 1). Only three sites, in the region of Stockholm, were located in catchments with more than 25% urban land. Otherwise, the median urban area was around 2%. Land use data on the catchments were extracted from the CORINE Land Cover datasets (European Commission 1993). The number of point sources upstream from the monitoring site was correlated to the size of the watershed area and varied from 0 to 24. The density of the point sources ranged from zero- to 0.075-point source per km^2^ catchment area (median 0.0035). There were 48 sites with no upstream point sources, while 17 sites had only one upstream point source. Data on the presence and number of point sources in the catchments were extracted from the Swedish pollution load compilation for the Baltic Sea (Swedish Environmental Protection Agency 2008).

The sites were sampled between 10 and 71 times each during the period 2010–2012, at a frequency ranging from every 2 weeks to every 4 months, and the samples were analyzed for SS, TP, turbidity, and electrical conductivity. All sites were sampled at least once each season of the year during the study period. Overall at all stations, more samples were taken during autumn (median of 5 times per station), while spring and summer were sampled a median of 3 times at each station. Winter was the least sampled season (median of 2 times per station).

In a case study, different techniques for estimating TP loads were compared using a stream draining an agricultural catchment (code name U8) within the national monitoring program for agricultural catchments (Kyllmar et al. 2014b). The catchment is located within the expanded area in Fig. 1 and covers approximately 6 km^2^, with 56% of the area dedicated to arable land. The long-term precipitation at the site is 539 mm, and the mean annual temperature is 5.9 °C (Kyllmar et al. 2014b). The dominant soil texture (according to the USDA classification) is clay. Water discharge in the stream was measured using a level gauge and a V-notch weir located upstream. Flow was measured every 30 s and then stored as daily mean values. The stream was sampled with two parallel methods: fortnightly grab sampling and flow-proportional composite sampling. The flow-proportional composite sampling was performed at a point situated 50 m upstream from the grab sampling point. Land use in the catchment areas adjacent to these sampling points is similar, with no contribution from any extra point source. The fortnightly composite sample consisted of sub-samples of approximately 20 mL, which were taken by a peristaltic pump every time a set volume of water passed the measurement station. The sub-samples were stored in a 10-L glass bottle, and a water sample was taken from the bottle every 2 weeks and sent to the laboratory for analysis (Kyllmar et al. 2014b). Turbidity was measured every 10 min between 4 July and 3 December 2012, using a field sensor (Hach-Lange SOLITAX sc and a SC-1000 controller), at approximately 10 m downstream from the point of the flow-proportional water sampling. The measurement range of the instrument is 0.001–4000 formazin nephelometric units (FNU) (Hach 2009). The instrument had a self-cleaning wiper device that cleaned the sensor before each measurement.

The data used in this study from the monitoring stations and the case study are available in Villa et al. (2019).

### Laboratory analyses

All analyses were performed with standard methods at the accredited laboratories of the Swedish University of Agricultural Sciences. Turbidity was measured using the standard method SS-EN ISO 7027 with the turbidimeter Hach 2100AN IS and expressed in FNU. Electrical conductivity was analyzed according to reference method SS-EN 27888-1 and expressed in mS m^−1^. Concentration of suspended solids was determined gravimetrically after extraction by suction filtering through a 1.2-μm glass fiber filter following a modified version of the standard method SS-EN 872:2005. The modification consists of analyzing the samples within 2 weeks instead of the standard 24 h and using a smaller pore size filter (0.2-μm membrane filter) in order to capture the small clay particles which dominate in the agricultural catchment. Total P was analyzed using the standard method SS-EN ISO 6878:2005, modified to adapt to the specific characteristics of the instrument Autoanalyzer 3 through the Bran*Luebbe Method No. G-175-96. Samples containing less than 4 μg dissolved reactive P (DRP) were analyzed following Bran*Luebbe Method No. G-175-96 Rev. 2 with a Bran*Luebbe Autoanalyzer 3. All other samples were pre-filtered (filter pore diameter 0.4 μm) to efficiently retain the colloidal clay particles and analyzed for DRP following the method ISO 15923-1:2013 using a Thermo Scientific™ Gallery™ Discrete Analyzer. Particulate phosphorus (PP) was determined as the difference between TP and DRP. Negative values of PP, ranging from − 2 to − 1 μg L^−1^, were detected in 1% of samples (30 out of 2358). These were due to measurement uncertainty and were set to zero for data analysis.

### Statistical analysis and calculations

The use of turbidity and conductivity as a surrogate for SS and TP was examined in the comprehensive study of the 108 monitoring sites through simple linear regression. The variables were log-transformed to fulfil the assumption of normal distribution of residuals. A non-parametric Spearman correlation test was used to assess whether a higher coefficient of determination (*r*^2^) in the turbidity-SS relationship was correlated with a percentage of agricultural land in a catchment. A non-parametric test was chosen because neither of the variables followed a normal distribution after log transformation. The impact of point sources on the relationship between turbidity/conductivity and TP was studied through analysis of variance (ANOVA) by comparing groups either with or without any point sources in the catchments.

In the case study on the agricultural monitoring catchment, TP loads were calculated from the fortnightly grab samples, flow-proportional composite samples, and high-frequency sensor measurements of turbidity. Conductivity was not measured in the case study, due to breakdown of the sensor. For the fortnightly grab samples, TP loads were estimated using calculated daily concentrations, which were estimated by linear interpolation of the fortnightly samples. The calculated daily concentrations were then multiplied by the daily discharge recorded values (HELCOM 2015; OSPAR Commission 2014). For the composite samples, TP loads were obtained for 2-week periods by multiplying cumulative flow over the period by the actual concentration for that period. For the high-frequency sensor measurements, TP loads were estimated by first calculating TP concentrations from turbidity values using the regression equation established from the grab samples in the same stream. Daily transport was then calculated by multiplying the estimated daily mean TP concentration by the daily discharge. Uncertainty in the TP loads calculated from high-frequency turbidity measurements was estimated using the lower and upper 95% prediction intervals of the turbidity (from high-frequency measurements) to TP (from manual sampling) regression. Baseflow index was calculated as the ratio of the baseflow values to the total stream flow values (Gustard et al. 1992). The calculation was performed using the time series from 1994 to 2012. The index ranges from 0 to 1 and is used to describe the hydrology at the catchment. Catchments with baseflow index close to 1 have a higher contribution of groundwater to streamflow, while catchments with low baseflow index are characterized by a larger contribution of overland and near-surface lateral flow. The software JMP 14.0 from SAS was used for the statistical analysis and calculations.

## Results and discussion

### Turbidity, SS, and TP concentrations in streams

The range of turbidity, SS, and TP reflected the fact that the selected streams covered a variety of conditions, from clear pristine waters to more polluted waters (Table 1). Compared with long-term losses from Swedish monitoring agricultural catchments (Kyllmar et al. 2014b), the maximum value of SS and TP was above the 90th and 75th percentiles, respectively.

**Table 1:** Concentrations of turbidity, suspended solids (SS), total phosphorus (TP), and conductivity measured at 108 stream monitoring sites in Sweden during 3 years (2010–2012)

Variable	Sampling method	*N*	Min.	Median	75th percentile	90th percentile	Max.
Turbidity (FNU)	Manual	2060	0.18	4.4	11.0	24.0	327.0
		1659^a^	0.18	5.5	14.0	28.0	327.0
SS (mg L^−1^)	Manual	2060	1.00	6.8	13.7	21.9	309.3
		1658^a^	1.00	5.4	11.4	21.5	309.3
TP (μg L^−1^)	Manual	2357	1.0	26.0	54.0	89.2	2222.0
		1955^a^	1.0	31.0	62.0	95.4	2222.0
Conductivity (mS m^−1^)	Manual	2357	0.62	5.9	28.25	46.5	115.0
		1955^a^	0.62	8.7	33.1	49.8	115.0

^a^Excluding the clear-cut forested area of Balån (six sites)

### Estimating SS from turbidity in streams

**Fig. 2:**
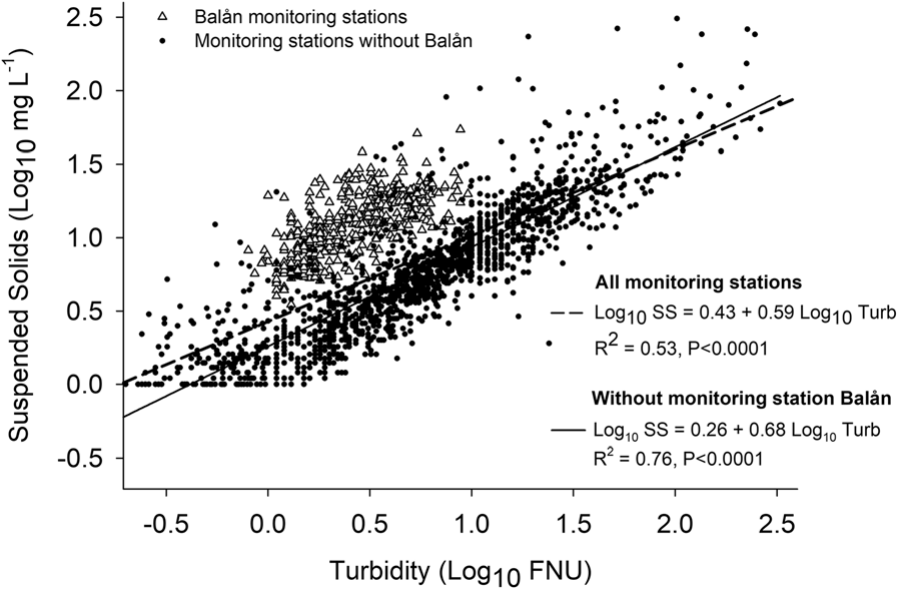
Linear regression between suspended solids and turbidity for all individual measurements from the 108 monitoring stations (*n* = 2061) and for samples excluding the samples from monitoring stations of Balån. Samples from the newly clear-cut forested area of Balån are represented by unfilled triangles

The general relationship between turbidity and SS considering all individual grab samples from all sites (*n* = 2061) produced an *r*^2^ value of 0.53 (Fig. 2). When all the individual samples were plotted, two groups of samples were identified. One of the groups had a higher content of SS in relation to the turbidity value, meaning that the water samples had less ability to intercept light compared with other samples (Fig. 2). Most of these samples came from the clear-cut forested area of Balån, part of the Balsjö Catchment Study (Löfgren et al. 2009). The monitoring stations of Balån are those located farthest north in Fig. 1. When these samples were excluded from the analysis, *r*^2^ increased to 0.76 (Table 2). For only the samples from Balån (*n* = 402), a lower *r*^2^ was obtained (*r*^2^ = 0.40, *P* < 0.0001). Previous studies in this geographical area have reported an increase in dissolved organic carbon (DOC) concentrations in boreal streams after forest clear cutting (Schelker et al. 2014). Dissolved organic material causes water discoloration, which can reduce the recorded turbidity due to the light-absorbing dissolved substances (Gippel 1995; Bright et al. 2018). This could be a possible explanation for the deviating behavior of samples from the newly clear-cut forested area.

**Table 2:** Results from linear regression (*y* = *α* + *βx*) between suspended solids (SS) and turbidity and between total phosphorus (TP) and turbidity, for 3 years (2010–2012) at a range of 108 stream monitoring stations in Sweden

No. of stations	No. of water samples	Linear regression term (*y*)	*x*	*α*	*β*	*r*^2^	RMSE	95% CI *β*	95% CI *α*
108	2060	Log SS	Log turbidity	0.43	0.59	0.53	0.30	0.56–0.61	0.41–0.45
102^a^	1658	Log SS	Log turbidity	0.26	0.68	0.76	0.22	0.66–0.70	0.24–0.27
108	2356	Log TP	Log turbidity	1.09	0.60	0.75	0.22	0.59–0.62	1.08–1.10
	2356	Log TP	Log turbidity, log conductivity	0.98	0.51 (turbidity); 0.17 (conductivity)	0.77	0.21	0.49–0.52 (turbidity); 0.15–0.19 (conductivity)	0.97–1.00
102^a^	1955	Log TP	Log turbidity	1.10	0.61	0.78	0.22	0.60–0.62	1.10–1.11

All regressions were significant at *P* < 0.0001

*RMSE* root mean square error, *CI* confidence interval

^a^Excluding the newly clear-cut forested area of Balån

**Fig. 3:**
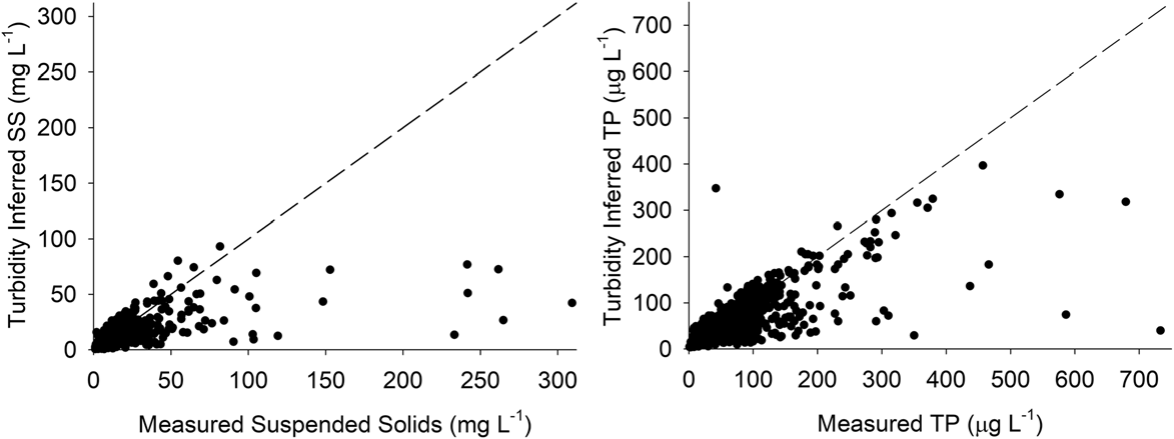
(Left) turbidity-based (modeled) suspended solids (SS) compared with measured concentrations (mg L^−1^). (Right) turbidity-based (modeled) total phosphorus (TP) compared with measured concentrations (μg L^−1^). The dashed line in both graphs represents the 1:1 line. The regression equation used to calculate SS from turbidity values was as follows: log_10_ SS = 0.26 + 0.68 log_10_ turbidity. The regression equation used to calculate TP from turbidity values was as follows: log_10_ TP = 1.09 + 0.60 log_10_ turbidity

The calculated SS values obtained were compared against the measured values (Fig. 3, left). Greater deviations from the 1:1 line were observed with higher measured SS concentrations, which did not correspond to higher turbidity values. Underprediction of high values of measured SS has been observed by others (e.g., Minella et al. 2008) and could be related to the effect of aggregation caused by an increase in organic matter. Larger aggregated particles have low light-scattering capacity and hence produce lower turbidity values for a given SS concentration (Slaets et al. 2014). The high values of measured SS in our study were found in samples from different sites, different regions, and different months of the year. The majority of the other samples from such sites were close to the 1:1 line, and the deviations from the 1:1 line were exceptions. To improve the understanding of the turbidity-SS relationship, more samples with high SS values would be needed.

**Fig. 4:**
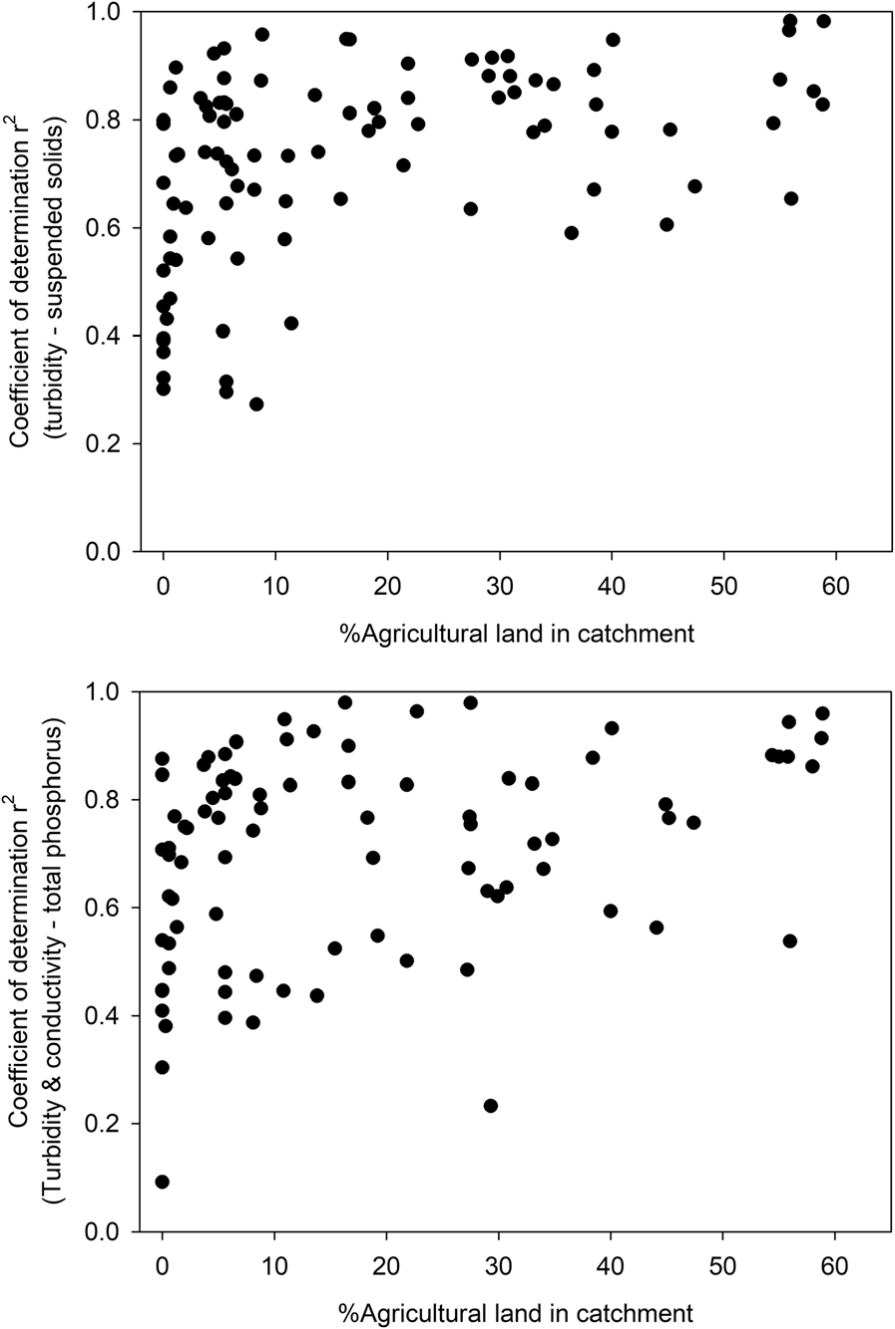
Variation of the coefficient of determination (*r*^2^) with the % agricultural land in the catchment, for the relationship between turbidity and suspended solids (SS) (*n* = 94 sites out of 108) and between turbidity and conductivity and total phosphorus (TP) (*n* = 84 sites out of 108)

The site-specific relationship between turbidity and SS was significant (*P* < 0.05) for 87% of the sites (94 sites). Non-significant relationships resulted from sites located in predominantly forested catchments. In summary, the mean *r*^2^ of all individual significant regression equations was 0.72 (range 0.27–0.98), which was slightly lower than the value obtained for the general regression (0.76). The most common value was 0.78, slightly above the mean. Mean slope for all the individual regressions was 0.8 (range 0.4–2.4). Coefficients < 0.5 were found for 13 sites, and all but one of which were located in small headwater catchments. The dominant land use at these sites was forest, except for one site located in an urban catchment (> 50% urban land use). Smaller streams have a faster response to precipitation and snowmelt and have higher variability in sediment response than larger rivers (Duvert et al. 2011; Jones et al. 2012). More frequent sampling could be needed in these small headwater forest streams to get a better description of SS variability and to confirm whether the use of turbidity is appropriate in this type of stream. Higher *r*^2^ was moderately positively correlated with the percentage of agricultural land (Spearman *ρ* = 0.47, *P* < 0.0001) and weakly negatively correlated with the percentage of forest in the catchment (Spearman *ρ* = − 0.35, *P* < 0.001). Those catchments with > 27% of agricultural land all had coefficients > 0.6, being the median value 0.85 (Fig. 4).

**Fig. 5:**
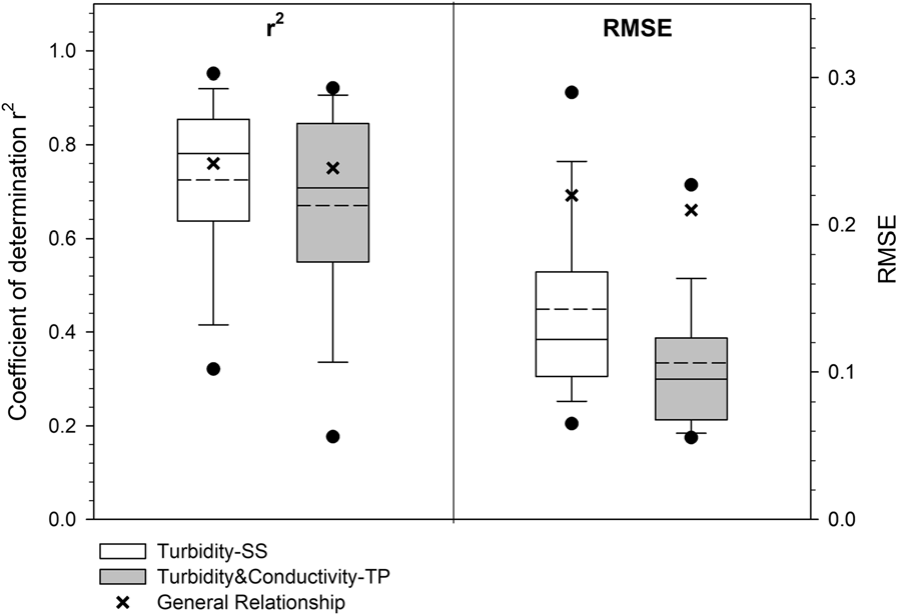
Distribution of *r*^2^ (left) and RMSE (right) values associated with the relationship between turbidity and suspended solids (SS) (*n* = 94 sites out of 108) and between turbidity and conductivity and total phosphorus (TP) (*n* = 84 sites out of 108). Lower and upper boundaries of the box indicate the 25th and 75th percentiles, respectively. The dots represent the 5th and 95th percentiles. The line inside the box is the median, and the dashed line is the mean. Only significant relationships were considered. The crosses represent the values of the general relationships

The number of observations made at each station and the *r*^2^ values were weakly correlated (Spearman *ρ* = − 0.24, *P* < 0.05). In fact, for the 13 stations with only 10 observations, *r*^2^ ranged from 0.1 to 0.95. The median *r*^2^ value for these stations was 0.74, which was very close to the overall *r*^2^ median value. The mean and median root mean square error (RMSE) for the site-specific regressions was 0.14 and 0.12, respectively, i.e., lower than the RMSE found for the general relationship (0.22) (see Table 2 and Fig. 5). These results indicate that most of the site-by-site regressions were more precisely predictive than the general relationship, which is in agreement with findings by Davies-Colley et al. (2014) for 77 rivers in New Zealand.

In summary, a general linear regression equation explaining the variation in SS with turbidity was applicable for most types of water bodies studied when streams draining clear-cut forested catchments at Balån were excluded. However, the error associated with predicting SS from turbidity for all the data was larger than that in more than 75% of the site-by-site regressions (Fig. 5). The range of the prediction error for single sites was also large. This implies that it is necessary to establish a site-specific relationship for any stream for which a turbidity sensor can potentially be used as a proxy for SS. Other studies have also shown that it is more appropriate to develop a specific regression for each stream and/or for dominant soil types (Grayson et al. 1996; Hayes et al. 2001), but this is not always possible due to a lack of data and constraints on resources. In this sense, streams where a good relationship is known (e.g., catchments with a high percentage of agricultural land) should be selected first for the use of turbidity sensors. For instance, the use of turbidity in small forest streams may not be advisable, due to the low turbidity values found and/or to the variation in origin and quality of suspended matter, which leads to weak relationships.

As in many other studies (e.g., Gippel 1995; Ruzycki et al. 2014), we used linear regression to predict SS from turbidity. Other types of regression, including polynomial or power relations, have also been used to explain the variation in SS with turbidity, especially for higher turbidity values or changing sediment sources (e.g., Sun et al. 2001; Zabaleta et al. 2007). When establishing a site-specific relationship, special efforts should be made to collect samples during high-turbidity events, to determine whether a linear or exponential model should be used. This was not possible in the present study, since we were restricted to the regular monitoring data.

### Estimating TP from turbidity and conductivity in streams

The relationship between turbidity and TP determined from grab samples was studied through linear regression. When all individual observations were included, *r*^2^ was 0.75 (*P* < 0.0001) (Table 2). Excluding the sites from the clear-cut forested area of Balån, *r*^2^ only slightly increased to 0.78 (*P* < 0.0001).

The site-specific turbidity-TP relationship was significant for 78% of the sites (84 sites), with a mean *r*^2^ value of 0.62. When conductivity was introduced as a second explanatory variable, the mean *r*^2^ increased to 0.67 (88 sites with significant regressions). At 25% of the sites, *r*^2^ was higher than 0.84 (Fig. 5). At 79 stations (73% of the total 108 stations), the turbidity-SS and turbidity-TP relationships were both significant. A greater variation in *r*^2^ (range 0.10–0.94) was found for the turbidity-TP relationship than for the turbidity-SS relationship (Fig. 5). The large spread in *r*^2^ values indicated that the sources and processes controlling the level of TP in different streams varied more than those controlling SS. Mean and median RMSE for the site-specific regressions was 0.11 and 0.095, respectively, i.e., lower than the RMSE found for the general relationship (0.21) (see Table 2 and Fig. 5). Over 90% of the site-specific regressions had a lower error than that obtained in the regression for the entire dataset (Fig. 5).

Higher *r*^2^ was weakly positively correlated with the percentage of agricultural land (Spearman *ρ* = 0.34, *P* < 0.001) and weakly negatively correlated with the percentage of forest in the catchment (Spearman *ρ* = − 0.33, *P* < 0.001). Overall, higher *r*^2^ values were found whenever there was a larger proportion of agricultural land in the catchment (Fig. 4), although this tendency is not as clear as in the case of the relationship turbidity-SS. The presence (or not) of point sources in the catchments did not result in important differences in either the strength of the turbidity-TP relationship or the concentrations of TP. In catchments with no point sources, *r*^2^ was significantly (*P* < 0.001) lower than that in catchments with point sources. The *r*^2^ value from ANOVA between the two groups (i.e., presence of point sources and no presence of point sources) was low (0.1), indicating that the differences were not very important. The concentration of TP was significantly higher (*P* < 0.01) in catchments with point sources, but low *r*^2^ (0.1) indicated that the difference could not be considered important. The relationship between turbidity and TP is reported to be influenced by the source of P (i.e., point or diffuse sources) (Viviano et al. 2014), by the flow conditions (Jordan et al. 2007), and probably most importantly, by the percentage of the different fractions of P, with a stronger relationship obtained when PP is the dominant form (Jones et al. 2011). The dominant soil texture in a catchment also influences this relationship. For example, in clay-dominated catchments, the share of P from point sources is small due to high levels of background losses, which are predominantly in PP form.

**Fig. 6:**
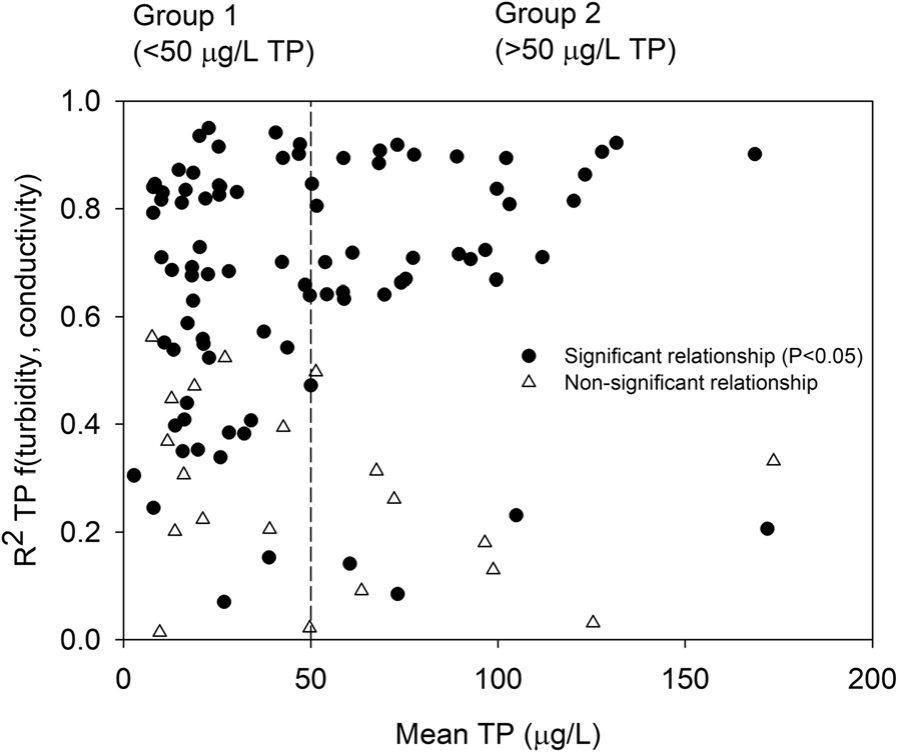
Variation in *r*^2^ (for the relationship between turbidity and total phosphorus (TP) and conductivity) with TP mean concentration at each site. Black dots represent significant relationships, while unfilled triangles represent non-significant relationships. The dotted line indicates a TP concentration of 50 μg L^−1^

In an effort to explain differences in *r*^2^ values, we explored the reasons behind the distribution at different TP concentrations. Through visual inspection based on the distribution of *r*^2^ values, two groups of *r*^2^ for the mean TP concentrations at each site were distinguished (Fig. 6). At lower TP concentration values, *r*^2^ values were evenly distributed (0–1), but from around 50 μg L^−1^, the distribution changed, with a larger proportion of *r*^2^ values above 0.6 (Fig. 6). In the first group (TP < 50 μg L^−1^), *r*^2^ values were evenly distributed (from 0 to 0.95) and mean *r*^2^ was 0.58. In the second group (TP > 50 μg L^−1^), mean *r*^2^ was slightly higher (0.62) and two main sub-groups, one with *r*^2^ > 0.6 and another with *r*^2^ < 0.4, could be distinguished. In both groups (1 and 2), low *r*^2^ (< 0.4) was mainly obtained for the non-significant relationships (Fig. 6). Overall, stronger relationships were found more consistently for high TP concentrations, which, in Sweden, often have a high particulate phosphorus content and are located in agricultural areas dominated by clay soils (Kyllmar et al. 2014b; Swedish Environmental Protection Agency 2009).

In summary, we did not detect correlations between *r*^2^ values and average chemistry or catchment characteristics. Since there is a strong correlation between TP and clay content in the monitored agricultural catchments in Sweden (Kyllmar et al. 2014b), it is possible that data on soil type within the catchments would help predict where turbidity has the highest potential for use as a proxy for TP. Unfortunately, no data on soil texture was available for most of the catchments from the present study. A more detailed study of stream hydrology and the amounts exported by each of the point sources, together with the stream flow, could help explain the differing strength of relationships between turbidity and conductivity and TP. Based on our results, the turbidity and conductivity-TP relationship works better overall in agricultural catchments with high TP losses and when the proportion of PP is large, irrespective of the source. The use of turbidity as a proxy for TP in predominantly forested areas is less justifiable. Even when the proportion of PP was high, the relationship obtained was weak, indicating that the character of the particulate matter (e.g., mineral, organic) might have an influence on the relationship between turbidity and the amount of particles present. For instance, Gippel (1995) observed that organic particles give 2- to 3-fold higher turbidity readings than mineral particles at a given concentration and particle size. Even if a good relationship between turbidity as a proxy for SS and TP can be established, some regular monitoring by grab sampling will still be needed, especially if the aim of the monitoring is to follow the effect of mitigation measures. As Bechmann and Øgaard (2013) point out, the introduction of a mitigation measure leading to a reduction in TP losses (e.g., change in fertilization strategy) may not necessarily translate into a reduction in SS. For that reason, it is important to sample both SS and P, to correct and recalibrate their relationship with turbidity when needed.

### Case study with in situ measurement of turbidity in an agricultural monitoring catchment

#### Relationship between turbidity and SS, TP, and PP in grab samples

Concentrations of SS from fortnightly grab sampling and high-frequency turbidity values for the same time point were significantly correlated (*r*^2^ = 0.87, *P* < 0.0001). The use of mean daily turbidity values instead of selected specific time values coinciding with grab sampling gave very similar values and relationships. Concentrations of TP and PP from grab sampling were also significantly related by linear regression (Table 3) to the high-frequency daily mean turbidity measurements (*r*^2^ = 0.91, *P* < 0.0001, for TP; *r*^2^ = 0.90, *P* < 0.0001, for PP).

**Table 3:** Regression equations (*y* = *α* + *βx*) between turbidity and suspended solids (SS) and between turbidity and total phosphorus (TP) in the agricultural monitoring catchment U8 in the period 4 July–3 December 2012

No. of observations	Linear regression term (*y*)	*x*	*α*	*β*	*r*^2^	RMSE	95% CI *β*	95% CI *α*
10	Log SS (grab sample)	Log turbidity (time-specific)	0.30	0.83	0.87	0.17	0.56–1.10	− 0.19 to 0.79
10	Log SS (grab sample)	Log turbidity (daily mean)	0.20	0.89	0.89	0.16	0.63–1.51	− 0.28 to 0.67
11	Log TP (grab sample)	Log turbidity (time-specific)	− 1.75	0.52	0.89	0.10	0.38–0.65	− 1.99 to − 1.51
11	Log TP (grab sample)	Log turbidity (daily mean)	− 1.81	0.55	0.91	0.09	0.42–0.68	− 2.04 to − 1.58
	Log PP (grab sample)	Log turbidity (time-specific)	− 2.06	0.59	0.87	0.12	0.42–0.77	− 2.37 to − 1.75
11	Log PP (grab sample)	Log turbidity (daily mean)	− 2.13	0.64	0.90	0.11	0.47–0.80	− 2.41 to − 1.84

Concentrations of SS and TP were analyzed in the grab samples, and turbidity was measured by an in situ sensor. All regressions were significant at *P* < 0.0001

*RMSE* root mean square error, *CI* confidence interval

#### In situ measurements compared with the analyzed water samples and modeled values

**Fig. 7:**
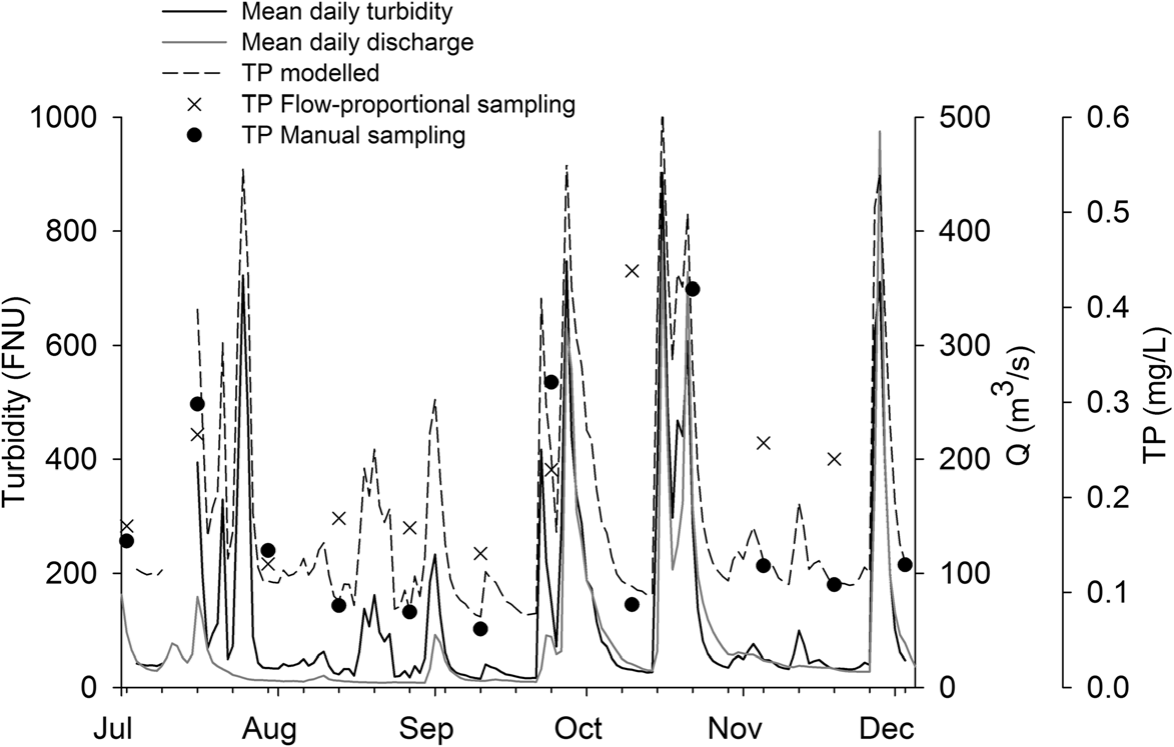
Temporal variation in mean daily turbidity (black line) and mean daily discharge (gray line) at the outlet of the agricultural monitoring catchment U8 in the period 4 July–3 December 2012. Concentrations of total phosphorus (TP) are represented by dots (fortnightly grab sampling) and a cross (end of a 2-week period of flow-proportional sampling). The modelled TP concentrations calculated from high-frequency turbidity measurements are represented by the dashed line

The majority of sediments and particle-associated P are transported during storm events, so failing to capture high-flow peaks can lead to underestimation of SS and P loads (Jones et al. 2011). During the first half of the study period (July–September), flow was low, but during the second half (October–December), four large discharge episodes were recorded. These episodes coincided with peaks in turbidity (Fig. 7). Other turbidity peaks were recorded during the first half of the study period, at low flows, and could be attributed to small rain events during summer. Fortnightly grab sampling captured two of the high flow peaks but missed the other two. Mean and median concentrations of SS obtained from fortnightly grab sampling and those estimated from turbidity were similar (Table 4). The main difference was observed in the higher ranges (both the 90th percentile and the maximum), which were higher in the case of turbidity-based SS. For TP, the pattern observed was very similar, with the greatest difference in the maximum values. PP ranged from 0.03 to 0.34 mg L^−1^ (Table 4). It was the main fraction of P in 10 of the 11 samples, ranging from 49 to 74% of TP.

**Table 4:** Concentrations of suspended solids (SS), total phosphorus (TP), and particulate phosphorus (PP) from fortnightly grab sampling

	Turbidity (FNU)	SS (mg L^−1^)		TP (mg L^−1^)		PP (mg L^−1^)
	Sensor, in situ	Fortnightly grab sampling	Turbidity-based SS	Fortnightly grab sampling	Turbidity-based TP	Fortnightly grab sampling
*N*	147	11	147	11	147	11
Mean	119.8	89	97	0.17	147	0.12
Min.	15.5	0	18	0.061	0.07	0.03
Median	46.2	54	48	0.13	0.13	0.092
75th percentile	106.6	138	101	0.30	0.21	0.21
90th percentile	389.1	314	320	0.40	0.42	0.31
Max.	902.7	336	677	0.42	0.67	0.34

Daily turbidity values from high-frequency measurements and concentrations of SS and TP were modeled based on high-frequency turbidity. Measurements in the agricultural monitoring catchment U8 in the period 4 July–3 December 2012 are shown

#### Biweekly loads of TP using three different methods

**Fig. 8:**
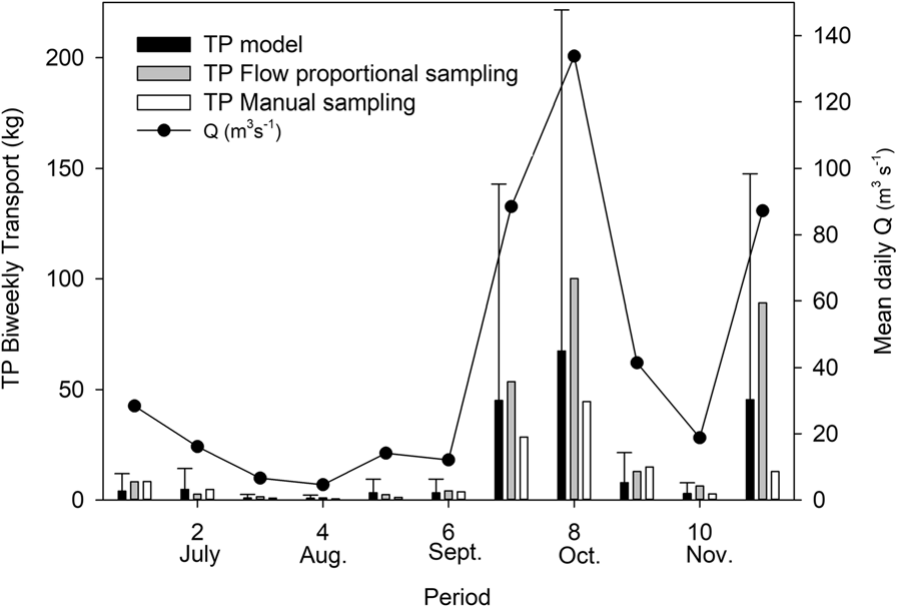
Fortnightly total phosphorus (TP) transport in the agricultural monitoring catchment U8 in the period 4 July–3 December 2012. Transport was calculated from grab sampling, flow-proportional concentration, and high-frequency turbidity measurements at the catchment outlet. The error bars in the modeled transport represent the 95th prediction interval. The line connecting dots indicates mean daily discharge

Total phosphorus loads were calculated based on fortnightly grab sampling, on flow-proportional sampling, and on modeled concentrations from the high-frequency turbidity measurements. The loads estimated with the three different sampling techniques were similar during low-discharge periods but differed during periods of high discharge (Fig. 8). During the high-discharge periods, TP loads calculated from flow-proportional concentrations were 19–51% larger than the modeled loads and 34–70% larger than the loads determined from grab sampling (95th prediction intervals). On a total basis for the whole study period, TP load in the agricultural monitoring catchment U8 calculated with flow-proportional sampling, high-frequency measurements, and grab sampling was 282 kg, 178 kg, and 123 kg, respectively.

Three different estimates of the TP load in the case catchment were obtained and compared. The relatively low loads from the grab sampling can be explained by the fact that the sampling missed two of the high-flow episodes, by sampling approximately 5 days before or after the discharge peak. Underestimation of TP load in fortnightly grab sampling is consistent with other findings for clay-dominated catchments in Sweden (Kyllmar et al. 2014a). This shows that it is essential to complement grab fortnightly sampling with other sampling techniques (e.g., flow-proportional sampling) to obtain more representative annual load estimates.

The discrepancy between the loads from flow-proportional sampling and high-frequency measurements (e.g., turbidity measured by sensor) is not as straightforward to explain. The relationship between TP and turbidity was linear for all the grab sampling data, where the value of the latter ranged up to 503 FNU, but in the high-frequency measurements, it ranged up to 1000 FNU. If the relationship between TP and turbidity levels off at higher values, that would lead to underestimation of the load from turbidity measurements at higher values of turbidity. The study did not include the period between January and June because the equipment was unable to measure during winter due to freezing. During these months, and especially during March and April, large flow events usually take place in the catchment due to melting snow. In future studies, it is important to cover these large events to include higher turbidity values in the load estimations. It is also possible that real TP values are even higher than those obtained by flow-proportional sampling. When the Swedish flow-proportional method was applied to high-frequency in situ data on TP for three streams in Ireland, it was concluded that the median estimated load was 80–96% of that based on in situ analysis (Cassidy et al. 2018). Another possible explanation for the discrepancies between the load estimates in the present study was that the different types of sampling (i.e., suction pipe, sensor, and grab sampling) took place at different locations in the stream, which could lead to errors due to the vertical and horizontal heterogeneity of SS (Horowitz 2013). Horowitz et al. (1990) observed that the type of sampler and the location and depth of sampling in a stream cross section affect SS and associated trace element concentrations and, consequently, load calculations. Suspended solids are composed of particles of different sizes and densities, and thus their distribution in the stream cross section is not necessarily homogeneous, which could lead to misrepresentation of P if, e.g., only small-sized and more P-enriched particles are sampled. Large rivers are more sensitive to variations in suspended solids in their cross section (Horowitz et al. 1990; Bouchez et al. 2010; Guinoiseau et al. 2016), but this issue might also be important in small streams such as that used in our case study.

Baseflow index in the catchment was low (0.3) which shows that the catchment behavior is flashy, with a rapid response to intense rainfall. There is a high proportion of clay soils in the catchment, and therefore, the contribution of the groundwater sources is low. Johnes (2007) observed that, when sampling infrequently, the uncertainty in the load estimations is higher for systems with a significant quickflow hydrological response than for systems with a higher baseflow index. In order to reduce uncertainty in the load estimates, more samples would be needed, especially at high values of turbidity (Fig. 8). The number of grab samples used in the present study was low (11), and therefore, more samples need to be used in future studies. Sensor measurements need to be complemented with manual sampling to calibrate/recalibrate the turbidity-TP relationship. Taking a grab sample during peak concentrations is a challenge due to the short duration of peak events, often just a few hours, but is very important in order to establish an accurate relationship between turbidity and TP. This can be solved by using telemetry-based infrastructure for continuous recording and transmission of flow data. This would provide access to data at any time; e.g., an alert could be sent when a high-flow event is occurring.

## Conclusions

Turbidity was found to be a good predictor of SS and TP, with a general relationship for most stream types. However, the error related to predicting SS and TP from turbidity for all the data was larger than that for the site-specific regressions in 75% and 90% of the cases, respectively. For single sites, there was a large range in how well turbidity could predict SS and TP (*r*^2^ ranged from 0.27 to 0.98 and from 0.10 to 0.94, respectively). Thus, a specific relationship based on grab samples should be established before a decision on deploying a sensor at a site is taken. There was a good relationship between SS and turbidity for catchments with a larger percentage of agricultural land. The relationship between TP and turbidity was stronger for catchments with high TP concentrations. These catchments in Sweden often have a high proportion of agricultural land, which is valuable, considering that most P export occurs from agricultural areas and appropriate mitigation measurements need to be focused there.

The case study showed that fortnightly grab sampling runs the risk of underestimating sediment and nutrient loads, as it fails to capture the highly temporal dynamics of these constituents. During the study period, fortnightly grab sampling only captured half of the high flow peaks. Flow-proportional sampling better captures SS and P loads but does not provide information on the short-term variations in concentrations needed for process evaluation. The TP loads estimated from grab sampling and modeled values were 34–70% and 19–51% smaller, respectively, than the loads from flow-proportional concentrations. To improve estimations of TP loads, more samples at high turbidity values should be taken. Turbidity should also be measured during a longer period in order to capture most of the large flow peaks. In the present case, for instance, large flow snowmelt peaks were not covered.

High-frequency measurements of turbidity by sensors in streams have great potential to increase knowledge about the sources and dynamics of SS and TP in streams. They can capture changes in concentrations not detected by flow-proportional sampling and provide more accurate estimates of TP loads than grab sampling. In addition, they offer the possibility to study within-channel processes and observe changes in water quality as a result of nutrient mitigation efforts over time. More accurate assessment of P loads derived from sensor data can also improve the calibration of models used to calculate the rates, retention, and source apportionment of P in river basins. Prior to establishing sensor measurements as a monitoring strategy, the variation in SS and TP in stream cross sections needs to be studied, to avoid misrepresentation of these constituents. In addition, to obtain a reliable relationship, intense sampling covering the whole flood hydrograph is needed. Climate change is expected to increase water flow and increase the incidence of strong high flows, increasing losses of SS and P. High-frequency measurements of turbidity as a surrogate for SS and P would provide valuable information for future projections and for quantifying the uncertainty in those projections.
